# Conserved Function of ACYL–ACYL CARRIER PROTEIN DESATURASE 5 on Seed Oil and Oleic Acid Biosynthesis between *Arabidopsis thaliana* and *Brassica napus*

**DOI:** 10.3389/fpls.2017.01319

**Published:** 2017-07-25

**Authors:** Changyu Jin, Dong Li, Chenhao Gao, Kaige Liu, Shuanghui Qi, Shaowei Duan, Zixiong Li, Jingyun Gong, Jianjun Wang, Jiangbo Hai, Mingxun Chen

**Affiliations:** State Key Laboratory of Crop Stress Biology for Arid Areas and College of Agronomy, Northwest A&F University Yangling, China

**Keywords:** AAD5, seed oil, stearic acid, oleic acid, *Arabidopsis thaliana*, Brassica napus

## Abstract

Previous studies have shown that several ACYL–ACYL CARRIER PROTEIN DESATURASE (AtAAD) members in *Arabidopsis thaliana* are responsible for oleic acid (C18:1) biosynthesis. Limited research has been conducted on another member, AtAAD5, and its paralog BnAAD5 in the closely related and commercially important plant, *Brassica napus*. Here, we found that *AtAAD5* was predominantly and exclusively expressed in developing embryos at the whole seed developmental stages. The *aad5* mutation caused a significant decrease in the amounts of oil and C18:1, and a considerable increase in the content of stearic acid (C18:0) in mature seeds, suggesting that AtAAD5 functioned as an important facilitator of seed oil biosynthesis. We also cloned the full-length coding sequence of *BnAAD5-1* from the A3 subgenome of the *B. napus* inbred line L111. We showed that ectopic expression of *BnAAD5-1* in the *A. thaliana aad5-2* mutant fully complemented the phenotypes of the mutant, such as lower oil content and altered contents of C18:0 and C18:1. These results help us to better understand the functions of AAD members in *A. thaliana* and *B. napus* and provide a promising target for genetic manipulation of *B. napus*.

## Introduction

Seed fatty acids (FAs) and FA-derived complex lipids not only provide nutrients for humans and livestock (Li et al., 2006; Graham, 2008), but also serve as raw materials for industries and biofuel production (Durrett et al., 2008; Lu et al., 2011). Biosynthesis of seed oil is under the control of multiple genes, and occurs in plant cells in three steps (Baud et al., 2008; Itabe, 2010; Chapman and Ohlrogge, 2012). The first step is the production of pyruvate and other substances during glycolysis. Catabolysis of pyruvate and other substances leads to the FA precursor acetyl-CoA, which results in biosynthesis of C16-18 FAs in plastids. In the second step, FA derivatives are formed at acyl chains. FA formation occurs in the cytoplasm after chain elongation and desaturation of most C16-18 FAs from the first step. Finally, triacylglycerols are formed to store the new oil in oil bodies.

There are six main types of FAs in seed oil from *A. thaliana*: palmitic acid (C16:0), stearic acid (C18:0), oleic acid (C18:1Δ^9^), α-linoleic acid (C18:2), α-linolenic acid (C18:3), and eicosenoic acid (C20:1^Δ11^); many minor FAs also exist in *A. thaliana* (<3 mol% each). The ACYL–ACYL CARRIER PROTEIN (ACP) DESATURASE (AAD) members have specificity for the acyl chain length of the particular substrate and generate double bonds between particular carbon atoms (Kachroo et al., 2007; Bryant et al., 2016). They are the only enzymes catalyzing the conversion of C18:0 into C18:1 in plant cells, and thus their activity primarily controls the saturated to monounsaturated FAs ratio (Kachroo et al., 2007).

The *A. thaliana* genome contains seven *AtAAD* genes, including SUPPRESSOR OF SALICYLIC ACID INSENSITIVE2 (*AtSSI2/AtFAB2*, AT2G43710), *AtAAD1* (AT5G16240), *AtAAD2* (AT3G02610), *AtAAD3* (AT5G16230), *AtAAD4* (AT3G02620), *AtAAD5* (AT3G02630), and *AtAAD6* (AT1G43800) (Kachroo et al., 2007; Bryant et al., 2016). AtSSI2, AtAAD1, AtAAD3, AtAAD4, and AtAAD5 have all been found to desaturate C18:0-ACP at the Δ^9^ position, and AtSSI2 and AtAAD3 were also shown to have specific activity on C16:0-ACP (Kachroo et al., 2007). Expression of *AtSSI2* occurs in the endosperm and embryo (Le et al., 2010). Stearic acid (C18:0) is accumulated in the *ssi2* mutant in seed and vegetative tissues. This accumulation indicates reduced Δ^9^ desaturation, because it occurs by depleting C18:1Δ^9^ (Lightner et al., 1994). AtAAD1 is strictly localized in the embryo (Le et al., 2010), and the *aad1* mutation causes a significant increase in C18:0 and a decrease in C18:1Δ^9^ contents in the embryo (Bryant et al., 2016). *AtAAD2* is most strongly expressed in the peripheral endosperm, and *AtAAD3* is most strongly expressed in the chalazal endosperm (Le et al., 2010). The *aad2* mutants exhibit an approximately 30% reduction in the levels of C18:1Δ^9^ plus C20:1Δ^13^, while the levels of C18:1Δ^9^ plus C20:1Δ^13^ in *aad4* mutants are not significantly changed in the endosperm and seed coat (Bryant et al., 2016). AtAAD3, which functions independently of AtSSI2, is responsible for the biosynthesis of C16:1Δ^9^, C18:1Δ^11^, and C20:1Δ^13^ in the endosperm and seed coat (Bryant et al., 2016). Taken together, the specific expression patterns and effects of AtSSI2 and AtAAD1-4 on seed FA biosynthesis have been gained a lot in *A. thaliana*. In addition, *AAD* genes occur in several plant species, and have been previously isolated and/or characterized (Shanklin and Somerville, 1991; Thompson et al., 1991; Cahoon et al., 1996, 1997; Lindqvist et al., 1996; Cahoon and Browse, 1998; Whittle et al., 2005). However, little is currently known about AtAAD5, and its paralog BnAAD5 in *Brassica napus*, the commercially important close relative of *A. thaliana*.

In the current study, we found that AtAAD5 is specifically expressed in the embryo during seed development in *A. thaliana*. We demonstrated that AtAAD5 promotes oil and C18:1 biosynthesis in *A. thaliana* seeds. We also cloned and functionally characterized *B. napus AAD5-1* (*BnAAD5-1*), showing that it exhibits a conserved role with AtAAD5 in regulating seed FA accumulation when expressed in *A. thaliana*.

## Materials and Methods

### Plant Materials and Growth Conditions

The Col-0 ecotype was used as the wild type *A. thaliana* control, and the mutants were *aad5-1* (SALK_129779C), and *aad5-2* (SALK_035968C) in the Col-0 background. All *A. thaliana* plants were grown at 22°C with a 16 h/8 h light/dark photoperiod, which has been reported in detail previously (Duan et al., 2017). The *B. napus* inbred line L111 was maintained in the greenhouse of South Campus, Northwest A&F University, China. T-DNA mutant were genotyped using specific primers (Supplementary Table S1).

### Gene Cloning of *BnAAD5-1* from *B. napus*

Primers were designed to amplify the *BnAAD5-1* gene based on the full-length coding domain sequence of *BnAAD5-1* (GenBank Number XP_013735719.1). Developing seeds were used as a source of total RNA to synthesize template complementary DNA (cDNA). Seeds were collected from the *B. napus* inbred line L111 15 days after pollination. We used the pMD18-T vector for cloning (TaKaRa Bio, Dalian, China), and eight single colonies were picked randomly and sequenced by Sangon Biotechnology (Shanghai, China). Cloning primers are listed in Supplementary Table S1.

### Plasmid Construction

To obtain the construct of *35S:BnAAD5-1*, the amplified full-length coding regions of *BnAAD5-1* were digested with Xma I and Spe I and then were cloned into pGreen-35S; this was driven by the 35S promoter. To construct *pAtAAD5:GUS*, the 824-bp *AtAAD5* genomic region including a 283-bp promoter region, ATG, and a 538-bp region downstream of the ATG start codon in sequence was amplified and then cloned into pHY107 (Liu et al., 2007). Plasmid construction primers are listed in Supplementary Table S1.

### Generation of *A. thaliana* Transgenic Plants

The *pAtAAD5:GUS* and *35S:BnAAD5-1* constructs were transformed into *Agrobacterium tumefaciens* GV3101 and were used to transform *A. thaliana* wild type and *aad5-2* plants, respectively, and the floral dip method was utilized (Clough and Bent, 1998). We used Basta^®^ selection and genotyping to confirm that plants were transgenic until T3 homozygous lines were obtained.

### Morphological Observation of Mature Seeds

Mature *A. thaliana* seeds were randomly selected from major inflorescences, specifically from siliques in the basal region, and photographed using an OLYMPUS SZ 61 stereomicroscope.

### Seed FA Measurement

Mature *A. thaliana* seeds for FA determination were collected from siliques in the basal region of the major inflorescences of 16 individual plants sown in different pots arranged in a randomized block design. Seed FA determination was conducted as previously described (Poirier et al., 1999; Chen et al., 2012). In brief, seeds were infused into the methanol solution containing 1 M HCl at 80°C for 2 h, which would convert FAs into the corresponding methyl esters. Then, FA methyl esters were extracted with the hexane, and were subsequently quantified by a gas chromatograph (GC-2014; Shimadzu).

### Gene Expression Analysis

Total RNA samples were isolated from *A. thaliana* young siliques or *B. napus* developing seeds with the MiniBEST Plant RNA Extraction Kit (TaKaRa) and their corresponding cDNA samples were biosynthesized with PrimerScript RT (TaKaRa). Reverse transcription-PCR (RT-PCR) and quantitative RT-PCR (qRT-PCR) were conducted for three biological replicates. SYBR Green Master Mix (TaKaRa) was utilized for qRT-PCR analysis. The *A. thaliana* house-keeping gene *AtEF1aA4* was regarded as an internal control. Primers used for the RT-PCR and qRT-PCR analyses are listed in Supplementary Table S1.

## Results

### Analysis of *AtAAD5* Expression Pattern

**FIGURE 1:**
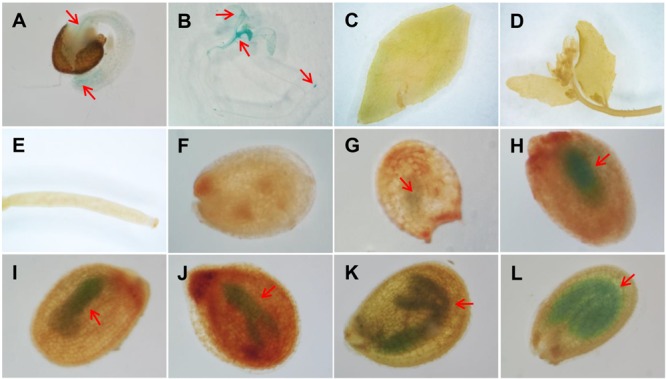
The expression pattern of *AtAAD5* as determined by GUS staining of the representative *A. thaliana pAtAAD5:GUS* line. **(A,B)** seedlings 1 **(A)** and 5 **(B)** days after germination; **(C)** rosette leaves; **(D)** cauline leaves and flowers; **(E)** siliques 3 days after pollination; **(F–L)** developing seeds at different stages (**F**: zygote; **G**: globular; **H–J**: liner cotyledon; **K**: bending cotyledon; **L**: mature green cotyledon). Red arrow indicates the position of GUS signal.

Previous RT-PCR results showed that *AtAAD5* was widely expressed in *A. thaliana* tissues, including leaves, stems, roots, flowers, and siliques (Kachroo et al., 2007). To better investigate the spatiotemporal expression pattern of *AtAAD5*, we obtained 19 independent lines of *pAtAAD5:GUS* from a wild type background. GUS staining patterns were similar among most of the lines; therefore, one representative line was used for GUS staining analysis. The result showed that *AtAAD5* was expressed in several tissues, including hypocotyl vascular bundles (**Figure 1A**), root tips (**Figures 1A,B**), cotyledons (**Figure 1B**), and young expanding true leaves (**Figure 1B**). Notably, *AtAAD5* was highly present in developing embryos at different stages (**Figures 1G–L**). However, no GUS staining was observed in other tissues, such as expanded true leaves (**Figure 1C**), cauline leaves (**Figures 1D**), flowers (**Figure 1D**), young siliques (**Figure 1E**), seed coats (**Figures 1F–L**), or endosperms (**Figures 1F–L**). These results suggested that *AtAAD5* controls seed traits mainly occurring in the *A. thaliana* embryo at the whole seed developmental stages (Baud et al., 2002; Fait et al., 2006; Graham, 2008; Baud and Lepiniec, 2009).

### AtAAD5 Promotes Oil and Oleic Acid Biosynthesis in Seeds

**FIGURE 2:**
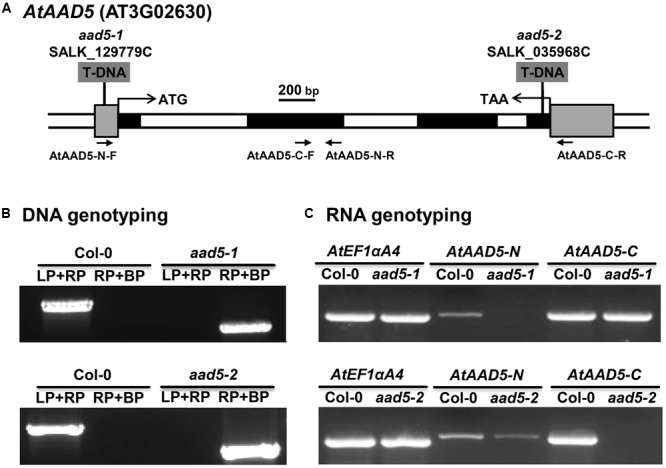
Molecular identification of the *A. thaliana aad5* mutation. **(A)** Structure of the *AtAAD5* gene indicating the position of the T-DNA insertions in SALK_129779C (*aad5-1*) and SALK_035968C (*aad5-2*) mutants. The coding and untranslated regions of *AtAAD5* are represented by black and gray boxes, respectively, and introns and other genomic regions are represented by open boxes. Translation start site (ATG) and stop codon (TAA) are indicated. **(B)** PCR-based DNA genotyping of the two mutants. LP and RP refer to the *AtAAD5* gene specific primers and BP refers to T-DNA right-border primer given in Supplementary Table S1. **(C)** Detection of N- and C- terminal transcripts of *AtAAD5* in the two mutants by RT-PCR using primer pairs indicated in **(A)**. *AtEF1αA4* was amplified as an internal control.

*AtAAD5* was previously screened by Kachroo et al. (2007) for T-DNA insertion mutants, but they did not obtain homozygous lines. In this study, we successfully obtained two T-DNA insertion mutants SALK_129779C and SALK_035968C from the Col-0 ecotype in the 5′ untranslated region and the exon of *AtAAD5*, respectively, from the Arabidopsis Biological Resources Center (ABRC), which were designated *aad5-1* and *aad5-2*, respectively (**Figure 2A**). The genotyping PCR result indicated the presence of the two homozygous mutants (**Figure 2B**). The RT-PCR result showed that the N- and C-terminal *AtAAD5* transcripts were not detected in *aad5-1* and *aad5-2* mutants, respectively (**Figure 2C**). More PCR product was amplified by C-terminal primers compared to N-terminal primers for the Col-0 RNA samples (**Figure 2C**), suggesting that the PCR amplification efficiency of the C-terminal primers is higher than that of the N-terminal primers. Notably, the C-terminal *AtAAD5* transcript in *aad5-1* was almost as strong as the wild type, which needs further investigation (**Figure 2C**).

**FIGURE 3:**
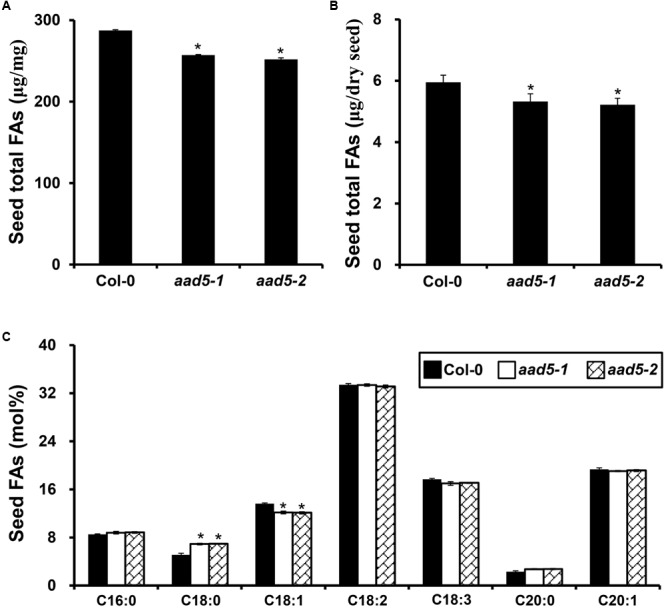
Effect of *AtAAD5* on *A. thaliana* seed FA biosynthesis. **(A)** Comparison of seed total FA content (μg/mg) between wild type (Col-0) and *aad5* plants. **(B)** Comparison of seed total FA content (μg/seed) between the wild type (Col-0) and *aad5* plants. **(C)** Comparison of contents of major seed FA compositions between the wild type (Col-0) and *aad5* plants. Asterisks indicate significant differences in the seed total FA content **(A,B)** and the contents of major seed FA compositions **(C)** compared to that in the wild type (two-tailed paired Student’s *t*-test, *P* ≤ 0.05). DW, dry weight. Values are means ± SD (*n* = 5). Error bars indicate standard deviation.

To explore the biological function of seed FA accumulation, we used mature seeds from wild type and *aad5* plants to determine the contents of major FAs. The result showed that the seed oil content was much lower in *aad5* mutants than in the wild type seeds (**Figures 3A,B**). In *aad5* seeds, there was a significant increase in the amount of C18:0 and a significant decrease in the C18:1 content (**Figure 3C**), suggesting that AAD5 plays a role in the desaturation of C18:0-ACP. However, we did not observe clear differences among morphological traits of seeds, including color of the seed coat, the size of the seed, or the dry weight of the seed **(Supplementary Figure S1)** between mature seeds of wild type and *aad5* plants. These results suggested that AtAAD5 promotes seed oil and oleic acid biosynthesis in the *A. thaliana* embryo.

### Analysis of BnAAD5-1 Sequence

**FIGURE 4:**
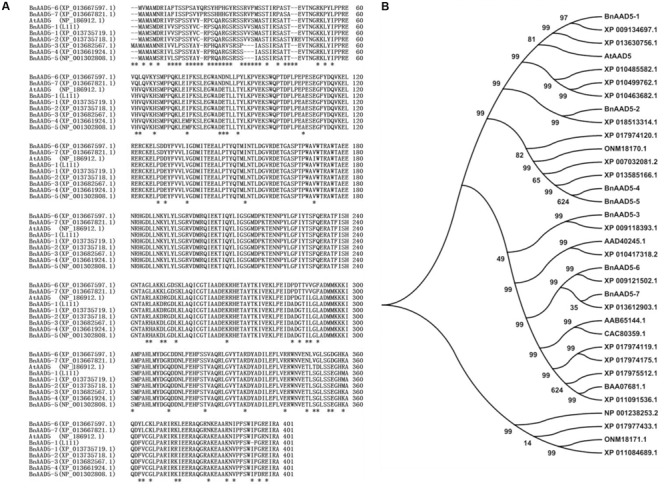
Sequence analysis of AAD5 proteins from *A. thaliana, B. napus*, and other oil-producing plant species. **(A)** Alignment of AAD5 protein sequences from *A. thaliana* and *B. napus*. The alignment was conducted using MUSCLE (http://www.ebi.ac.uk/Tools/msa/muscle/) and discrepant amino acids are indicated by asterisks. **(B)** Phylogenetic analysis of 34 AAD5 family proteins from 11 oil-producing plant species. A neighbor-joining tree (Jones–Taylor–Thornton model) was generated by MEGA6. A bootstrap analysis with 1,000 replicates was performed to assess the statistical reliability of the tree topology. The accession numbers corresponding to the species names are as follows: AtAAD5 (*A. thaliana*); BnAAD5 1-7 (*B. napus*); XP_009134697.1, XP_018513314.1, XP_009118393.1, and XP_009121502.1 (*B. rapa*); XP_013630756.1, XP_013585166.1, and XP_013612903.1 (*B. oleracea*); AAD40245.1 (*B. juncea*); ONM18170.1 and ONM18171.1 (*Zea mays*); XP_010499762.1, XP_010485582.1, XP_010463682.1, and XP_010417318.2 (*Camelina sativa*); BAA07681.1, XP_011091536.1, and XP_011084689.1 (*Sesamum indicum*); AAB65144.1 and CAC80359.1 (*Helianthus annuus*); NP_001238253.2 (*Glycine max*); XP_017977433.1, XP_017975512.1, XP_007032081.2, XP_017974119.1, XP_017974175.1, XP_017974120.1 (*Theobroma cacao*).

We named the seven *BnAAD5* paralogs from the *B. napus* genome *BnAAD5 1-7* (**Figure 4**). As shown in **Figure 4A**, the cloned BnAAD5-1 (L111) sequence from the *B. napus* inbred line L111 was the same as the BnAAD5-1 protein sequence (XP_013735719.1) of the *B. napus* cultivar Zhongshuang11 (ZS11). *BnAAD5-1* is located on the A3 subgenome of the *B. napus* cultivar ZS11, and our sequence results suggested that we had cloned *BnAAD5-1* from the L111 A3 subgenome. BnAAD5-1 (L111) was also predicted to share 96% amino acid sequence identity with AtAAD5 (**Figure 4A**).

We performed a phylogenetic analysis to investigate the evolutionary relationship between BnAAD5-1 and 33 AAD5 proteins from 11 oil-producing plant species. The analysis indicated that BnAAD5-1 is most related to the three AAD5 sequences, including BrAAD5 (XP_009134697.1) from *B. rapa*, BoAAD5 (XP_013630756.1) from *B. oleracea*, and AtAAD5 from *A. thaliana* (**Figure 4B**).

### BnAAD5-1 Fully Rescues the FA Phenotype of *A. thaliana aad5-2* Seeds

**FIGURE 5:**
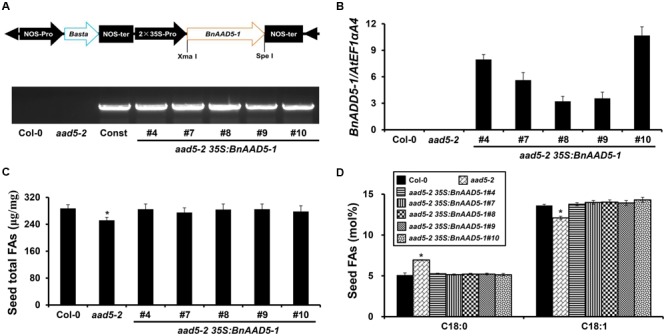
Effect of *BnAAD5-1* on seed FA accumulation when expressed in the *A. thaliana aad5-2* mutant. **(A)** Schematic diagram of the constitutive expression cassette of the *BnAAD5-1* gene in the binary vector *pGreen 2 × 35S* (Top figure) and PCR-based DNA genotyping of *aad5-2 35S:BnAAD5-1* transgenic plants with the specific primers: 35S_Pro and BnAAD5-1_R1 (Bottom figure). RB, right border; LB, left border; NOS-pro, nopaline synthase promoter; NOS-ter, nopaline synthase terminator; Basta^®^, glyphosate; 35S-pro, CaMV 35S promoter, Const: *35S:BnAAD5-1* construct. **(B)** Comparative analysis of *BnAAD5-1* expression by qRT-PCR in the wild type (Col-0), *aad5-2*, and *aad5-2 35S:BnAAD5-1* plants. Expression levels were normalized against the expression of the internal control, *AtEF1αA4*. Error bars indicate standard deviation. **(C)** Quantitative comparison of total FA content between the wild type (Col-0), *aad5-2*, and *aad5-2 35S:BnAAD5-1* seeds. Asterisks indicate statistically significant differences in total FA content of *aad5-2* seeds compared to that of wild type seeds (two-tailed paired Student’s *t-*test, *P* ≤ 0.05). Values are means ± SD (*n* = 5). Error bars indicate standard deviation. **(D)** Quantitative comparison of FA compositions of C18:0 and C18:1 between the wild type (Col-0), *aad5-2*, and *aad5-2 35S:BnAAD5-1* seeds. Asterisks indicate statistically significant differences in contents of FA compositions of *aad5-2* seeds compared to that of wild type seeds (two-tailed paired Student’s *t-*test, *P* ≤ 0.05). Values are means ± SD (*n* = 5). Error bars indicate standard deviation.

To further elucidate the function of *BnAAD5-1* in seed FA biosynthesis, we over-expressed it in the *A. thaliana aad5-2* mutant, using the construct *35S:BnAAD5-1* (**Figure 5A**). A total of 23 independent T1 transgenic plants were obtained following Basta^®^ selection, and five independent transgenic lines (*aad5-2 35S:BnAAD5-1* T3) were confirmed by PCR amplification of the *BnAAD5-1* gene with the specific primers 35S_Pro/BnAAD5-1_R1 (**Figure 5A**; Supplementary Table S1). Expression of the *BnAAD5-1* gene in these transgenic plants was measured by qRT-PCR, and was determined to be highest in the transgenic line *aad5-2 35S:BnAAD5-1#10*, whereas its expression was not detected in the wild type or *aad5-2* plants (**Figure 5B**). We observed that ectopic expression of *BnAAD5-1* fully rescued *aad5-2* seed phenotypes, such as lower oil content (**Figure 5C**) and altered contents of C18:0 and C18:1 (**Figure 5D**). Although the *aad5-2 35S:BnAAD5-1#8* transgenic line showed the lowest expression of *BnAAD5-1*, the contents of total FAs, C18:0, and C18:1 were close to those of other transgenic plants (**Figures 5B–D**). This indicated that BnAAD5-1 regulates seed FA accumulation in a dose-independent manner when overexpressed in *A. thaliana*. These results together suggested that BnAAD5-1 has a similar function to AtAAD5.

## Discussion

The increase and optimization of FA composition in oil-producing plant seeds is the most important objective for breeders. Several of the seven *AtAAD* genes, including *AtSSI2, AtAAD1, AtAAD2, AtAAD3*, and *AtAAD4*, have been functionally identified for seed FA biosynthesis in *A. thaliana* (Kachroo et al., 2007; Bryant et al., 2016). However, little is known about the role of AtAAD5, and its paralog BnAAD5 in seed FA accumulation. Our results provide two major lines of evidence for a conserved and important role for AAD5 in mediating total FAs accumulation in seeds and C18:1 accumulation in the embryo in both *A. thaliana* and *B. napus*.

First, the *aad5* mutation resulted in a considerable increase in the amounts of oil and C18:0, and a significant decrease in the C18:1 content in mature seeds (**Figure 3**). The expression of *AtAAD5* was stably observed in developing embryos, but not in the endosperm and seed coat, during the whole seed developmental stages (**Figure 1**). C18:1 FA mainly exists in the forms of C18:1Δ^9^ and C18:1Δ^11^ in the *A. thaliana* embryo and endosperm plus seed coat, respectively, and C18:1Δ^11^ FA only accounts for less than 1 mol% of total FAs in the embryo (Bryant et al., 2016). The previous study showed that AtAAD5 preferentially desaturates C18:0-ACP substrate at the C9 position (Kachroo et al., 2007). These results together suggested that AtAAD5 plays an important role in controlling the conversion of C:18-ACP to C18:1Δ^9^ in the *A. thaliana* embryo. Traits of seeds including color of the coat, size of the seed, and weight of the seed were not altered in *aad5* mutants, which is consistent with the fact that *AtAAD5* was not expressed in seed coat and endosperm (**Figure 1F–L**). Intricate regulatory networks control FA accumulation in seeds. These networks also require coordinated development of three distinct seed tissues: embryo, endosperm, and seed coat. Therefore, the disruption of the structural gene *AtAAD5* might disturb seed embryo development, causing lower seed oil accumulation (**Figures 3A,B**). AtAAD5 and AtAAD1 are most closely related based on phylogenetic analyses of the AtAAD family; they are 82% identical at the amino acid level (Kachroo et al., 2007). Consistently, AtAAD1 and AtAAD5 showed similar functions on the conversion of C18:0-ACP to C18:1Δ^9^ in the *A. thaliana* embryo (**Figure 3C**; Kachroo et al., 2007). It is worth mentioning that AtAAD1 negatively affects C18:2 biosynthesis (Kachroo et al., 2007), whereas AtAAD5 has no significant effect on the accumulation of C18:2 and other major seed FAs except for C18:0 and C18:1 (**Figure 3C**). These results indicated that the two genes have some differences in the regulation of seed FA biosynthesis in the *A. thaliana* embryo.

Second, ectopic expression of *BnAAD5-1* cloned from the A3 subgenome of the *B. napus* inbred line L111 in the *aad5-2* mutant fully rescued altered seed FA contents of the mutant (**Figure 5**). This strongly suggested that BnAAD5-1 exhibits a conserved role with AtAAD5 in regulating seed FA accumulation when expressed in *A. thaliana*. However, no obvious differences were observed in the contents of oil, C18:0, and C18:1 in seeds between *aad5-2 BnAAD5-1* overexpressors and the wild type control (**Figure 5**). This indicates that the alterations caused by reduced expression of AtAAD5 and increased expression of BnAAD5-1 in *A. thaliana* do not simply mirror each other. *Arabidopsis thaliana* and *B. napus* are both part of Cruciferae, and there are three *A. thaliana* loci in the *B. rapa, B. oleracea*, and *B. nigra* genomes (Kowalski et al., 1994; Osborn et al., 1997; Lagercrantz, 1998; Haberer et al., 2006). *Brassica rapa* and *B. oleracea* hybridize to create *B. napus* (Parkin et al., 1995; Osborn et al., 1997). During *B. napus* evolution, there was a high frequency of rearrangement, fusion, and deletion of chromosomes (Lagercrantz, 1998), which led to, on average, 2-8 paralogs in the *B. napus* genome for each gene locus in *A. thaliana* (Osborn et al., 1997; Cavell et al., 1998). Here we found a single copy of *AtAAD5* in the *A. thaliana* genome as expected, and seven putative *BnAAD5* paralogs in the *B. napus* genome (**Figure 4A**). Our previous study showed that BnTOP1α-1 from the inbred L111 line has lost 4 amino acid stretches, compared with BnTOP1α-1 (XP_013685667.1) from ZS11, which collectively correspond to 130 amino acids (Gao et al., 2017). However, the cloned BnAAD5-1 from the inbred L111 line has the same sequence as BnAAD5-1 (XP_013735719.1) from ZS11 at the protein level (**Figure 4A**), and is most related to BrAAD5 (XP_009134697.1), BoAAD5 (XP_013630756.1), and AtAAD5 (**Figure 4B**). Saturated FA quantity in *B. napus* has been increased by seed-specific antisense repression of one *BrAAD* gene from *B. rapa* (Knutzon et al., 1992). These results indicate that AAD5 might be conserved during evolution of the cruciferous species (*A. thaliana, B. rapa, B. oleracea*, and *B. napus*).

In summary, this study is the first to identify that an AtAAD member, AtAAD5, is responsible for converting C18:0-ACP to C18:1 and promoting oil accumulation in the *A. thaliana* embryo. In addition, we showed that BnAAD5-1 has a conserved function with AtAAD5 in regulating seed FA accumulation when it is expressed in *A. thaliana*. *Brassica napus* is grown as a crop primarily for its seed oil. The identification and manipulation of key *B. napus* genes controlling seed oil and FA accumulation are of fundamental importance for agricultural production. These results suggest that BnAAD5-1 can be used as a promising target to genetically manipulate *B. napus* and other oil-producing plants to improve the amounts of seed oil, C18:0, and C18:1.

## Author Contributions

CJ and DL carried out the experiments. CJ and CG analyzed the data. KL, SQ, SD, ZL, JG, and JW assisted with doing the experiments. MC conceived and designed the experiments. MC and CJ wrote the manuscript. DL, CG, and JH helped to draft the manuscript and revise the manuscript. All authors read and approved the final manuscript.

## Conflict of Interest Statement

The authors declare that the research was conducted in the absence of any commercial or financial relationships that could be construed as a potential conflict of interest.

## Supplementary Material

FIGURE S1 Characterization of seed traits from the wild type (Col-0), *aad5*, and *aad5-2 35S:BnAAD5-1* plants. **(A)** Microscopy of mature seeds that were selected randomly from wild type, *aad5*, and *aad5-2 35S:BnAAD5-1* plants. Bars: 500 μm. **(B)** Quantitative comparisons of seed size (length and width) between the wild type, *aad5*, and *aad5-2 35S:BnAAD5-1* plants. **(C)** Quantitative comparison of dry weight of seeds between the wild type, *aad5-2*, and *aad5-2 35S:BnAAD5-1* plants. Error bars indicate standard deviation. No significant differences in seed size **(B)** or seed weight **(C)** were observed between wild type and *aad5* or *aad5-2 35S:BnAAD5-1* (two-tailed paired Student’s *t*-test, *P* ≤ 0.05). Values are means ± SD (*n* = 5). Error bars indicate standard deviation.
