# Supplementary figures and images for: Conserved Function of ACYL–ACYL CARRIER PROTEIN DESATURASE 5 on Seed Oil and Oleic Acid Biosynthesis between Arabidopsis thaliana and Brassica napus
Source: Front Plant Sci. 2017 Jul 25;8:1319. doi: 10.3389/fpls.2017.01319 (PMC5524766; doi:10.3389/fpls.2017.01319)

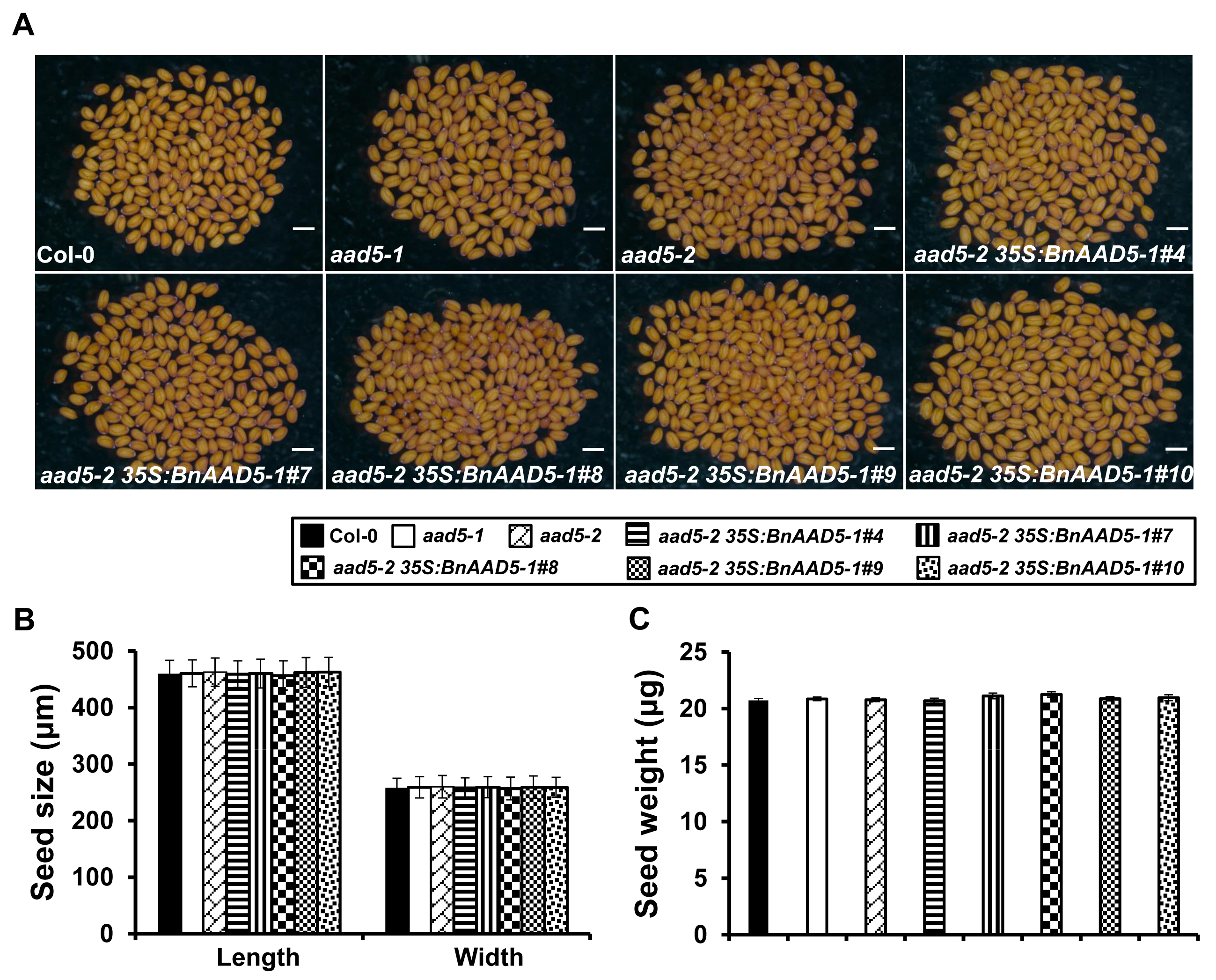

Supplement: FIGURE S1 — Characterization of seed traits from the wild type (Col-0), aad5, and aad5-2 35S:BnAAD5-1 plants. (A) Microscopy of mature seeds that were selected randomly from wild type, aad5, and aad5-2 35S:BnAAD5-1 plants. Bars: 500 μm. (B) Quantitative comparisons of seed size (length and width) between the wild type, aad5, and aad5-2 35S:BnAAD5-1 plants. (C) Quantitative comparison of dry weight of seeds between the wild type, aad5-2, and aad5-2 35S:BnAAD5-1 plants. Error bars indicate standard deviation. No significant differences in seed size (B) or seed weight (C) were observed between wild type and aad5 or aad5-2 35S:BnAAD5-1 (two-tailed paired Student’s t-test, P ≤ 0.05). Values are means ± SD (n = 5). Error bars indicate standard deviation. [file Image_1.jpg]
